# National Gay Men’s HIV/AIDS Awareness Day — September 27, 2018

**DOI:** 10.15585/mmwr.mm6737a1

**Published:** 2018-09-21

National Gay Men’s HIV/AIDS Awareness Day (https://www.cdc.gov/features/ngmhaad/index.html) is observed on September 27, 2018, to direct attention to the ongoing and disproportionate impact of human immunodeficiency virus (HIV) infection and acquired immunodeficiency syndrome (AIDS) on gay, bisexual, and other men who have sex with men (MSM) in the United States. Whereas MSM represent approximately 2% of the U.S. population (*1*), in 2016 they accounted for 66.8% of new diagnoses of HIV infection; MSM who inject drugs account for an additional 3.0% (*2*). Among MSM with new diagnoses of HIV infection in 2016, 49.4% were aged 13–29 years, 38.2% were aged 30–49 years, and 12.4% were aged ≥50 years (*3*). During 2008–2016, the number of annual new diagnoses increased 3% per year among MSM aged 13–29 years, decreased 4% per year among MSM aged 30–49 years, and was stable among those aged ≥50 years.

CDC supports a range of efforts to reduce HIV infection among MSM. These include HIV prevention services that increase diagnosis of HIV infection (https://www.cdc.gov/hiv/group/msm/index.html), support the linkage and engagement of MSM in care and treatment, and reduce the risk for acquiring and transmitting HIV infection (https://www.cdc.gov/msmhealth; https://www.cdc.gov/hiv/group/msm/bmsm.html).
